# Supplementary material for: Development and validation of a health education module for parents of schoolchildren with overweight and obesity in the UAE
Source: PLoS One. 2026 Mar 17;21(3):e0344967. doi: 10.1371/journal.pone.0344967 (PMC12994848; doi:10.1371/journal.pone.0344967)
Supplement: Attachment — Submitted filename: Response to Reviewers.docx [file pone.0344967.s003.docx]

Dear academic editor and respected reviewers,

Thank you for taking the time to review this paper and for your thorough feedback. The comments you provided have been addressed below.

**Journal Requirements**

1. Please ensure that your manuscript meets PLOS ONE's style requirements, including those for file naming.

The style and format of the paper have been reviewed to make sure it meets the journal requirements. Minor format editing have been done to the title of the paper and the headings.

**Reviewers Comments to the Authors**

**Reviewer #2:**

“The manuscript addresses an important public health concern and presents a relevant and well-structured study. Overall, the paper is technically sound, the methodology is appropriate, and the findings are clearly presented. Below are my detailed comments:

Technical Soundness and Rigor: The study design, consisting of two phases (module development and validation), is appropriate and clearly described.

Statistical analysis was performed according to recommended standards, and results are presented transparently.

Data Availability: The authors have complied with PLOS ONE’s data availability policy. All relevant data are included within the manuscript tables and the supporting information files. This ensures reproducibility and transparency.

Presentation and Language: The manuscript is presented in a clear and intelligible manner, with standard scientific English throughout.

Strengths of the Manuscript: The study is culturally relevant and fills a gap in the literature by focusing on parental involvement in the UAE context.

The educational module is flexible and adaptable for different platforms (booklet, online, social media), which increases its applicability and potential impact.

The authors have been transparent in acknowledging the limitation that only content and face validity were tested, and they appropriately recommend further reliability and effectiveness studies.

Ethics and Compliance: Ethical approval and informed consent are clearly reported.”

**Response:**

Thank you for your kind and detailed feedback. The minor issues mentioned in your comment have been addressed as follows:

1. On page 13, “health massages” should be corrected to “health messages.”

The spilling mistake in “health messages” has been corrected (page 15).

2. A few sentences could be smoothed for readability (e.g., “step started with a background reading” → “the step began with a background literature review”).

The sentence has been modified as suggested (page 6 / line 113-114).

3. The abstract and conclusion include some repetition; these could be slightly condensed without loss of clarity.

The abstract and the conclusion have been rephrased and condensed to make them clearer and to avoid repetition (page 2, 24).

**Reviewer #3:**

“The article was reviewed under the title "Development and validation of a health education module for parents of schoolchildren with overweight and obesity in the UAE".

Overall, the focus of the study is good. However, I would like to offer several recommendations that authors may find useful in the process of revising their manuscript.”

**Response:**

Thank you for your feedback and the useful recommendations. Your comments have been addressed as follows:

1. Given the urgency of the issue, a review of previous research is needed.

A paragraph was inserted in the introduction section reviewing previous studies (page 4-5 / line 78-90).

2. Considering that it is stated at the end of the introduction that this article was written with the aim of filling an important gap in the literature, in addition to describing previous research, it is necessary to describe and introduce the existing gaps.

The gaps and the need for this study were also described in the new paragraph inserted in the introduction section to review previous studies (page 4-5 / line 78-90).

3. For better readability, it is necessary to express the overall workflow in the form of an algorithm, flowchart, or figure.

A flowchart has been added to the end of the methods section with a summary of the main phases of this study (page 10 / line 223-226). The actual figure is uploaded as a separate file.

4. Given that one of the stages of the work was the development of the health education module, it is necessary to explain the basic model.

A paragraph was added to the health education module development phase section under methods emphasizing the use of the health belief model in the designing of the module (page 6 / line 128-134).

5. Given the statistical nature of the work, a more comprehensive statistical analysis of the results is needed.

The description of the statistical analysis was indicated within the explanation of the used approaches of CVI and FVI. However, to make it clearer and more statistically formatted, a subheading (Data analysis) was added towards the end of the methods section, that explains the details of the conduction of data analysis and the calculations (page 9-10 / line 202-222).

6. The number of the statistical population is not specified correctly. Only the time frame for data collection is mentioned.

The number was specified within the methods of each validation. To correctly specify it, it was added under the study design and ethics at the beginning of the methods section (page 6 / line 110-111).

7. The article states that this method will serve as a comprehensive, high-quality educational tool for use in current and future research studies. In this regard, no comparison between the results obtained with previous research has been made to substantiate this claim.

The discussion section has been modified and integrated with previous research (page 20-23). A paragraph has been added as well with a comparison that substantiate the mentioned claim (page 23 / line 362-373).
