# Supplementary material for: Development and validation of a health education module for parents of schoolchildren with overweight and obesity in the UAE
Source: PLoS One. 2026 Mar 17;21(3):e0344967. doi: 10.1371/journal.pone.0344967 (PMC12994848; doi:10.1371/journal.pone.0344967)
Supplement: Attachment — Submitted filename: Comments.docx [file pone.0344967.s004.docx]

The article titled " Development and validation of a health education module for parents of schoolchildren with overweight and obesity in the UAE" was reviewed again.

All cases have been corrected by the respected authors, but in one case, " For better readability, it is necessary to express the overall workflow in the form of an algorithm, flowchart, or figure. " despite the authors' response, unfortunately the flowchart in question is not visible in the text of the article.
