# Supplementary material for: Development and validation of a health education module for parents of schoolchildren with overweight and obesity in the UAE
Source: PLoS One. 2026 Mar 17;21(3):e0344967. doi: 10.1371/journal.pone.0344967 (PMC12994848; doi:10.1371/journal.pone.0344967)
Supplement: Attachment — Submitted filename: Response_to_Reviewers_auresp_2.docx [file pone.0344967.s005.docx]

Dear academic editor and respected reviewers,

Thank you for taking the time to review the last revisions. Upon reviewing your letter, only one comment was found in the document attached to the letter, and it has been addressed below.

Comment:

1. The article titled " Development and validation of a health education module for parents of schoolchildren with overweight and obesity in the UAE" was reviewed again.

All cases have been corrected by the respected authors, but in one case, " For better readability, it is necessary to express the overall workflow in the form of an algorithm, flowchart, or figure. Despite the authors' response, unfortunately the flowchart in question is not visible in the text of the article.

Response:

Thank you for your comment. The flowchart was attached as a separate file that can be found in the file inventory of the submission as per the journal’s instructions regarding figures. However, in this case and for the respected reviewer to be able to see the flowchart, it has been added in the manuscript within the text (in addition to the separate file) (page 11).
