# Supplementary material for: Development and validation of a health education module for parents of schoolchildren with overweight and obesity in the UAE
Source: PLoS One. 2026 Mar 17;21(3):e0344967. doi: 10.1371/journal.pone.0344967 (PMC12994848; doi:10.1371/journal.pone.0344967)
Supplement: Attachment — Submitted filename: Comments_declet_2.docx [file pone.0344967.s006.docx]

The article titled " Development and validation of a health education module for parents of schoolchildren with overweight and obesity in the UAE " was reviewed again.

This revision has significantly improved the manuscript. The authors have satisfactorily addressed my concerns.
